# The temporal dedifferentiation of global brain signal fluctuations during human brain ageing

**DOI:** 10.1038/s41598-022-07578-6

**Published:** 2022-03-07

**Authors:** Yujia Ao, Juan Kou, Chengxiao Yang, Yifeng Wang, Lihui Huang, Xiujuan Jing, Qian Cui, Xueli Cai, Jing Chen

**Affiliations:** 1 Institute of Brain and Psychological Sciences, Sichuan Normal University, No. 5, Jing’an Road, Chengdu, 610066 China; 2 Faculty of Education, Sichuan Normal University, Chengdu, China; 3 Tianfu College of Southwestern University of Finance and Economics, Chengdu, China; 4 School of Public Affairs and Administration, University of Electronic Science and Technology of China, Chengdu, China; 5 Psychological Research and Counseling Center, Southwest Jiaotong University, Chengdu, China; 6 School of Education, Chengdu Normal University, Chengdu, China

**Keywords:** Cognitive ageing, Neural ageing

## Abstract

The variation of brain functions as healthy ageing has been discussed widely using resting-state brain imaging. Previous conclusions may be misinterpreted without considering the effects of global signal (GS) on local brain activities. Up to now, the variation of GS with ageing has not been estimated. To fill this gap, we defined the GS as the mean signal of all voxels in the gray matter and systematically investigated correlations between age and indices of GS fluctuations. What’s more, these tests were replicated with data after hemodynamic response function (HRF) de-convolution and data without noise regression as well as head motion data to verify effects of non-neural information on age. The results indicated that GS fluctuations varied as ageing in three ways. First, GS fluctuations were reduced with age. Second, the GS power transferred from lower frequencies to higher frequencies with age. Third, the GS power was more evenly distributed across frequencies in ageing brain. These trends were partly influenced by HRF and physiological noise, indicating that the age effects of GS fluctuations are associated with a variety of physiological activities. These results may indicate the temporal dedifferentiation hypothesis of brain ageing from the global perspective.

## Introduction

Resting-state functional magnetic resonance imaging (rs-fMRI) provides abundant information for large-scale brain bases of age-related cognitive changes^1^. Numerous studies have documented alterations with ageing in almost all functional networks with rs-fMRI^1,2^. The global signal (GS) of rs-fMRI, as the average signal of the whole brain, has a great impact on the functional brain organization from local neural activities to inter-regional connections^3^. The characteristics of GS varying with age, therefore, is a key to understanding the age-related functional brain organization, which has not yet been elucidated.

The debate about whether the GS reflects neural signal or physiological noise has lasted for over two decades^4^. Although early studies have found prominent artifacts (i.e. head motion, respiration) in the GS^5,6^, numerous recent studies have tied GS fluctuations to vigilance^7^, behavioral traits^8^, brain states^9^, and mental disorders^3,10^, suggesting that the GS conveys particular physiological, psychological, and pathological information^11^. A recent study found a close relationship between the GS of rs-fMRI and the global EEG signal at multiple frequency bands^12^. By contrast, another study causally demonstrated that the GS could be regulated by signals from the basal forebrain^13^. Besides, Tsvetanov et al. suggested that the age effect of blood oxygen level-dependent (BOLD) signal variability could be fully explained by cardiovascular and cerebrovascular factors^14^. Since the GS has both neural and non-neural origins, it is worth exploring whether the neural, vascular, and physiological factors contribute differently to the variation of GS with age.

It has been demonstrated that the BOLD signal as well as other brain signals has two major characteristics: scale-free and oscillation^15,16^. The former refers that the power of brain signals tends to fall off with increasing frequency following a power-law function, while the latter refers to the recurring pattern of brain activity that follows a particular temporal beat^15^. The scale-free characteristic of BOLD signal in local brain regions has been demonstrated to be suppressed by arousal, cognitive tasks, and ageing^17,18^. That is to say, the power of BOLD signal transferred from the lower frequency end to the higher frequency range to improve short-term brain functions^16^. On the other hand, two oscillations within the infra-slow frequency range (< 0.1 Hz) have been identified^19–22^. However, the functions of these oscillations haven’t been determined. The variance of these two characteristics of GS with ageing which may contribute to the physiological mechanism of BOLD signal, hasn’t been studied yet.

In the current study, we aimed to investigate the age effect of GS fluctuations (i.e., the scale-free and oscillation characteristics) during the adult lifespan, using a large-sample of rs-fMRI data. We tested the relationship between age and GS indices, such as the power of GS fluctuations and the coefficient of scale-free characteristic. The spectral centroid (SC) rather than peak frequency of each oscillation was adopted to be the representative frequency of each oscillation and its variance with age because there was no obvious peak for some subjects^23^. In addition, these indices were tested with less pre-processed data and de-convolved data to test the influence of physiological and neurovascular factors on GS fluctuations. Because local BOLD signal fluctuations have been reported to be increased and decreased with age in different brain regions and frequencies^24,25^, we hypothesized to find frequency-dependent rise and fall of GS fluctuations with age. These frequency-dependent effects may support the idea that brain signal fluctuations get faster with age^26–28^.

## Results

There was no significant correlation between mean GS and age (r = 0.04, *p* = 0.53 for original data, r = 0.06, *p* = 0.282 for de-convolved data, and r =  − 0.03, *p* = 0.62 for data without noise regression). Significantly negative correlations between the SD of GS and age were observed for original data (r =  − 0.47, *p* < 0.001), but dramatically reduced for data without noise regression (r =  − 0.14, *p* = 0.01), and disappeared for de-convolved data (r =  − 0.06, *p* = 0.312), suggesting that the decline of GS variability with age may be caused by multiple factors including neural activity, physiological noise, and neurovascular coupling.

**Figure 1:**
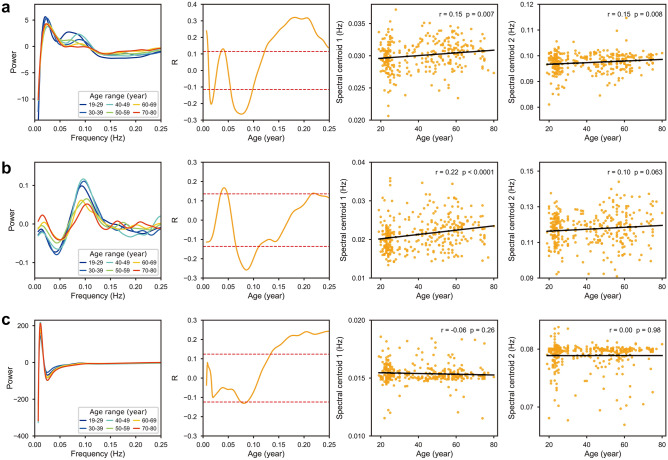
The variations of GS power and oscillation with ageing. (**a**) Original data. (**b**) De-convolved data. (**c**) Data without noise regression. Column 1: the power spectrum of GS after detrending. Each line represents the average power spectrum of subjects every 10 years. Column 2: the correlation between age and power at each frequency point. Red dashed lines indicate the thresholds of r with FDR correction (q < 0.05). Column 3 and 4: the correlations between age and SC for oscillation 1 and oscillation 2, respectively.

The GS power was significantly correlated with age in multiple frequency bands [0.007–0.01 Hz (r = 0.12–0.24), 0.013–0.022 Hz (r =  − 0.12 to − 0.20), 0.036–0.043 Hz (r = 0.12–0.13), 0.055–0.098 Hz (r =  − 0.12 to − 0.26), and 0.118–0.25 Hz (r = 0.12–0.32); FDR corrected, q < 0.05 corresponding r =  ± 0.12] for the original data (Fig. 1a, column 1 and 2). After HRF de-convolution, significant correlations (FDR corrected, q < 0.05 corresponding r =  ± 0.14) were found at 0.037–0.05 Hz (r = 0.14–0.17), 0.067–0.112 Hz (r =  − 0.14 to − 0.26), and 0.137–0.139 Hz (r = 0.14) (Fig. 1b, column 1 and 2). For data without noise regression, significant correlations (FDR corrected, q < 0.05 corresponding r =  ± 0.12) were found at 0.075–0.087 Hz (r =  − 0.13 to − 0.12) and 0.134–0.25 Hz (r = 0.12–0.24), respectively (Fig. 1c, column 1 and 2). A general trend was exhibited that the power with lower value tends to increase with age while the power with higher value tends to decrease with age. In addition, HRF de-convolution reduced correlations between GS power and age at the lower and higher frequency ends whereas physiological noise reduced those correlations within the infra-slow frequency range.

For the original data, the frequency ranges of oscillation 1 and oscillation 2 were 0.007–0.047 Hz and 0.047–0.149 Hz, respectively (Fig. 1a, column 1). The SCs of the two oscillations tended to shift to higher frequencies with age (Fig. 1a, column 3 and 4). For de-convolved data, oscillation 1 and oscillation 2 were located at 0.007–0.043 Hz and 0.043–0.195 Hz, respectively (Fig. 1b, column 1). The SCs of the two oscillations shifting to higher frequencies with age were also observed (Fig. 1b, column 3 and 4), indicating that this phenomenon cannot be interpreted by the effect of neurovascular coupling. In addition, the SC of oscillation 1 was moved to lower frequencies [t (321) =  − 43.3, *p* < 0.0001, Cohen’s d =  − 2.42] whereas that of oscillation 2 was moved to higher frequencies [t (321) = 46.0, *p* < 0.0001, Cohen’s d = 2.56] by HRF de-convolution. For data without noise regression, oscillation 1 and oscillation 2 were located at 0.007–0.025 Hz and 0.026–0.13 Hz, respectively (Fig. 1c, column 1). The frequencies of both oscillations were not changed by age (Fig. 1c, column 3 and 4), suggesting that the frequency shifting of SCs with age was counteracted by physiological noise. However, the SCs of oscillation 1 and oscillation 2 were both moved to lower frequencies [t (321) =  − 101.2, *p* < 0.0001, Cohen’s d =  − 5.65; t (321) =  − 73.6, *p* < 0.0001, Cohen’s d =  − 4.12] by physiological noise. These results suggested that the frequency shifting of SCs with age may be associated with neural activity, irrespective of the influences of neurovascular coupling and physiological noise on the frequency ranges of the two oscillations.

**Figure 2:**
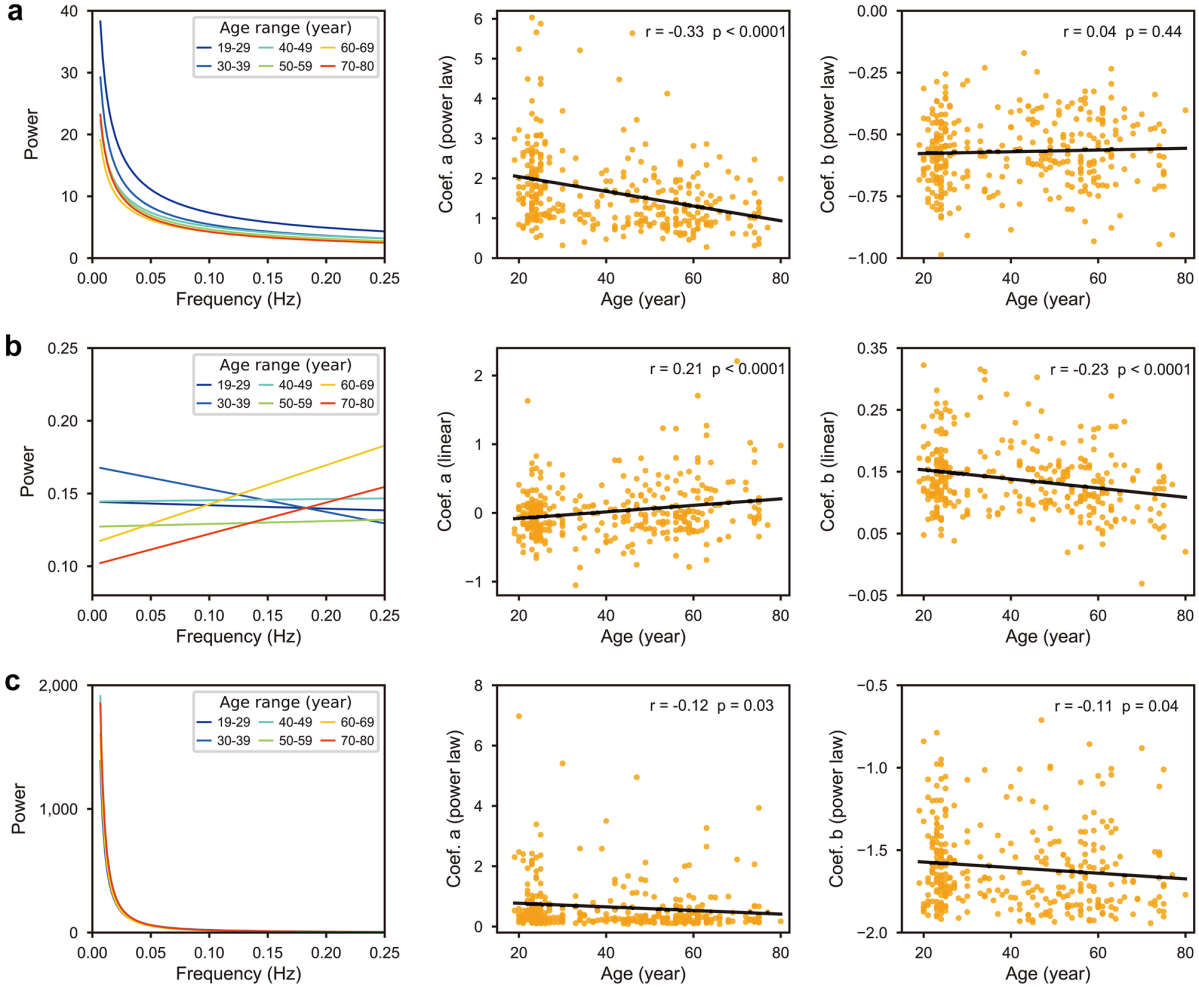
The relationship between age and the trend of power spectrum of GS. (**a**) Original data. (**b**) De-convolved data. (**c**) Data without noise regression. Column 1: the trend of GS. Each line represents the average trend of subjects every 10 years. Column 2 and 3: the correlation between age and coefficient *a* and* b* of trend functions, respectively.

The power-law trend of the original GS power spectrum was reduced with age (Fig. 2a, column 1), which was mainly determined by decreased coefficient *a* (the height of the power-law function; Fig. 2a, column 2) rather than *b* (the curvature of the function; Fig. 2a, column 3), indicating that brain ageing does not change the scale-free curve of GS power spectrum, but reduces the overall power especially in the lower frequency end. For the de-convolved data, the slope of linear trend (coefficient *a*) increased with age from negative to positive (Fig. 2b, column 1 and 2), while the intercept (coefficient *b*) decreased with age (Fig. 2b, column 3), suggesting that GS power transfers from lower frequency to higher frequency as brain ageing. For data without noise regression, coefficients *a* and *b* were both decreased with age to some extent (Fig. 2c), indicating a systematic decrease of the power-law trend. These results suggested that GS power transfers from lower frequencies to higher frequencies with age, irrespective of the influences of neurovascular coupling and physiological noise on the scale-free characteristic.

Finally, a significant positive correlation between the power of FD and age was found at 0.007–0.025 Hz (q < 0.05, FDR corrected). Both positive and negative correlations between GS power and age were observed within this frequency range for the original data, while no correlation was found for the de-convolved data and less preprocessed data. These evidence suggested that correlations between GS power and age cannot be explained by head motion.

## Discussion

The current results revealed GS fluctuations varied as ageing in three aspects: general power reduction, power transferring from lower frequencies to higher frequencies, and more even distribution of power across frequencies, which directly indicate the relationship between GS fluctuations and age during the adult lifespan for the first time. More importantly, these findings argue a temporal dedifferentiation interpretation of brain ageing. Age-related variations of GS fluctuations have both neural and non-neural origins. These variations of GS fluctuations with ageing are essential to understand altered functional organization as brain ageing.

To begin with, these findings are consistent with the general decline of local BOLD signal fluctuations with ageing in extensive regions^24,25^, which has been suggested to represent a less complex neural system with smaller dynamic range and attenuated ability to efficiently process ever-changing external stimuli^29^. The present findings demonstrated that the GS, as the average of local signals, shows the same trend as local signals. Garrett and colleagues have demonstrated that the decline of local BOLD signal fluctuations predicts age up to four times better than the mean BOLD signal^29^. The decline of brain signal (fMRI and EEG) fluctuations also appeared from newborn children to adults^30^. Combined with current findings, we suggest a general trend of low frequency power decline across the human lifespan.

Secondly, frequencies of the two oscillations increased with age. The same trend of BOLD signals was found in the first year of life^28^ as well as in resting-state brain networks during the adult lifespan^25^. Similar frequency transfer of EEG signal fluctuations from the slower range to the faster range with age has been reported during the first 20 years of life^26,27^. The migration of brain signal fluctuations to higher frequencies from resting-state to task-state was also observed in many task-based fMRI studies, which was suggested to reflect the brain expends more effort on immediately rapid tasks^16,17,31,32^.These evidence suggest that the migration of brain signal fluctuations to higher frequencies with age is a universal phenomenon throughout the lifespan, which may have something to do with the brain maintaining normal cognitive functions in the elderly.

Thirdly, the GS power was more evenly distributed in ageing brain, showing by (1) increased power with age if the power is lower in young people and vice versa and (2) power transferring from lower frequencies where the power is high to higher frequencies where the power is low. These phenomena were much similar to the spatial dedifferentiation of brain ageing, which argued that brain functions recruit more distributed rather than specialized brain regions in the elderly brain^33^. Analogously, we interpret the more evenly distributed power in elderly brain as temporal dedifferentiation. The spatiotemporal dedifferentiation may be of importance for preserving brain functions and preventing functional degeneration during brain ageing^34^.

Fourthly, besides the neural origin, the decline and temporal dedifferentiation of GS fluctuations with ageing were contributed by neurovascular coupling and vascular factors to some extent. Grinband et al. suggested that neurovascular coupling does not change significantly with normal ageing^35^, whereas Tsvetanov et al. argued that the age effect of BOLD signal variability can be fully explained by cardiovascular and cerebrovascular factors^14^. To reconcile previous inconsistent findings, West et al. investigated the HRF changes with healthy ageing based on large sample sizes and minimal analysis assumptions^36^. They observed increased time-to-peak and decreased peak amplitude in older compared to younger adults in sensory and motor regions. These major changes occurred within 20 s (corresponding to 0.05 Hz), which are in accordance with our findings that the frequency of oscillation 2 is higher and the power-age correlation around 0.05 Hz is greater after HRF de-convolution. However, the data included no measure of vascular health or of the vascular component of the BOLD signal, preventing us from detecting the influences of vascular factors on the correlation between age and GS fluctuations. According to previous findings^37,38^, we hypothesize that age-related vascular changes would affect GS fluctuations to some extent. Furthermore, many task-based fMRI studies have demonstrated that the BOLD signals after HRF de-convolution are closely associated with neural activity^39^. Therefore, temporal dedifferentiation of GS fluctuations with age may be contributed by neural, vascular, and neurovascular coupling factors embedded in the BOLD signals.

Finally, physiological noises such as head motion, white matter, and cerebrospinal fluid signals cannot be the cause of the age effects of GS fluctuations. Instead, they dramatically diminished these effects. Although Chen et al. discovered that resting-state networks formed by so-called physiological noise are highly overlapping with intrinsic networks^40^, our results suggest that physiological noise counteracts the effect of neural activity on the temporal dedifferentiation of GS fluctuations with age. It has been demonstrated that head motion^41^, respiratory^42^, and cardiac signals^43^ contain meaningful physiological and pathological information. Therefore, it is necessary to isolate different contributions of these components to brain ageing.

Some limitations remain. First, the age effect of physiological noise was indirectly tested due to the lack of those information in the open database. The actual contributions of different components (i.e. respiratory and cardiac signals) should be tested directly in future studies. Second, head motion parameters were strictly restricted and regressed out, which may eliminate motion-related physiological information^41^. Thus, the contribution of head motion to brain ageing warrants further studies. Third, the cognitive relevance of our results cannot be determined for the lack of cognitive measurements in this dataset. Given the close relationship between brain signal fluctuations and cognition in particular frequency bands^25^, our findings in multiple frequency bands may be associated with various cognitions which deserves in-depth studies. Fourth, subjects were not normally distributed amongst all ages which may influence the results of correlation analysis. Although the mean values of every ten years mirrored our main results (see column 1 of Figs. 1, 2) and a large sample size was used to enhance the robustness of the results, the current results should be verified in a more evenly distributed data. Finally, there were more females (n = 194) than males (n = 128) in the final analysis. We regressed out sex information and did not test the sex effect for it isn’t the major concern in the present study. However, the influence of sex on brain ageing is inconclusive and deserves further investigations^44^.

## Conclusion

We investigated GS fluctuations across the adult lifespan. The decline and temporal dedifferentiation of GS power with age were confirmed to be general patterns of brain ageing. These patterns may be driven by various physiological components. The temporal dedifferentiation extends the classical theory of spatial dedifferentiation in ageing brain and requires further verification.

## Methods

### Participants

A total of 492 adult volunteers (307 females, aged range 19 to 80 years) were recruited from Southwest University (SWU, China)^45^. The participants were primarily recruited through leaflets, online advertisements, and face-to-face propaganda. The exclusion criteria included the following: (1) MRI-related exclusion criteria, e.g., claustrophobia, metallic implants, Meniere’s Syndrome and a history of fainting within the previous 6 months; (2) current psychiatric disorders or neurological disorders; (3) use of psychiatric drugs within the three months prior to scanning; (4) pregnancy; or (5) a history of head trauma^45^.

The data collection was selected from a large dataset of individuals who participated in the ongoing brain imaging, creativity, and mental health data collection initiative. It was initiated in 2010, terminated in 2015, and shared to the scientific community in 2018^45^. The young sample (18–25 years) was enrolled as college students at Southwest University. Many of the middle-aged adults (26–40 years) were recruited from staff at Southwest University. The rest of the participants were recruited from communities close to the university campus. In addition, a part of the participants served as a control sample in a case-controlled study of a clinical population^45^.

### Ethics declarations

The project was approved by the Research Ethics Committee of the Brain Imaging Center of Southwest University, conducted in accordance with the World Medical Association Declaration of Helsinki. Written informed consent was obtained from each participant.

### Imaging acquisition and preprocessing

All rs-fMRI data were collected using a 3 T Siemens Trio MRI scanner (Siemens Medical, Erlangen, Germany) at the Brain Imaging Center of SWU. Subjects were asked to close their eyes, rest without thinking about any in particular, but refrain from falling asleep. Two hundred and forty-two volumes were acquired for each subject using the T2-weighted gradient echo planar imaging (EPI) sequence: 32 slices of 3 mm, slice ga* p* = 1 mm, TR/TE = 2000/30 ms, flip angle = 90°, field of view = 220 mm × 220 mm, resulting in a voxel with 3.4 × 3.4 × 4 mm^3^.

**Figure 3:**
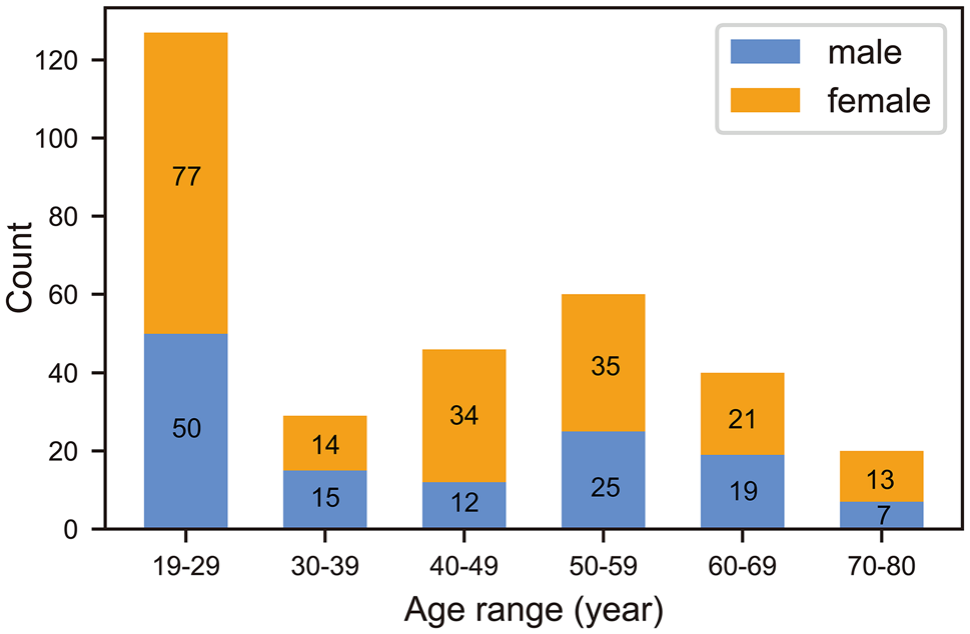
The distribution of participants based on age and sex. The exact number of each group is shown in the corresponding bar.

Image preprocessing was conducted using the Data Processing Assistant for Resting-State fMRI package (DPARSF, http://www.restfmri.net)^46^ according to steps in previous studies^5,47^: removing the first 12 volumes, slice timing and realignment. Subjects whose translational and rotational displacement exceeded 2.0 mm or 2.0° or mean frame-wise displacement (FD) exceeded 0.2 were excluded. The remaining sample included 322 subjects (194 females; mean age = 41.48, SD = 17.36). As shown in Fig. 3, there were more females than males (χ^2^ = 13.528, *p* < 0.001) and more subjects under 30 years than in other age ranges (χ^2^ = 137.988, *p* < 0.001). It has been demonstrated that using the EPI template, compared with anatomical images, in spatial normalization could amplify the statistical power of the sample^48^. Therefore, images were normalized to the standard EPI template, resampled to a 3 × 3 × 3 mm^3^ cube, and spatially smoothed (6-mm FWHM Gaussian kernel). The mean white matter and cerebrospinal fluid signals were extracted from relative regions defined by the EPI template. Linear trend, white matter, cerebrospinal fluid signals, and Friston 24 motion parameters were used as regressors to control the effect of head movement and non-neuronal information^49^.

### Power spectrum analysis of GS fluctuations

The GS was obtained by averaging signals over all gray matter voxels constrained by the binary automated anatomical labeling (AAL) 90 mask^50,51^. The Welch method with hamming window (window width 0.031 Hz, overlap rate 50%) was applied to transform time series into frequency domain^52^. Data were cutoff within 0.007–0.25 Hz for de-noising^53^. The power-law function *y* = *a* × *x*^*b*^ was applied to separate the fractal trend from oscillations because the original BOLD signal consisted of a scale-free trend and two oscillations^54^. Frequency boundaries of oscillations were determined by the local minima on the mean power density curve of all subjects^25^. For each subject, the SC of each oscillation was calculated within the defined frequency boundaries using Eq. (1), representing the center of gravity of the power spectrum within the given range of oscillation^23^

1 $$ SC = \frac{{\sum\nolimits_{{i_{1} }}^{{i_{2} }} {i \times f \times P(i)} }}{{\sum\nolimits_{{i_{1} }}^{{i_{2} }} {P(i)} }} $$

where *f* = 0.25/256 Hz, representing the width between two successive frequency points, $P(i)$ indicates the power at the $i{\text{th}}$ frequency point within $i_{1}$–$i_{2}$ Hz.

### Hemodynamic response function (HRF) de-convolution

The basic hypothesis underlying the BOLD signal is the convolution of neural events and neurovascular coupling^55^. In order to determine whether the neurovascular coupling is responsible for the relationship between GS power and age, the blind HRF de-convolution approach was performed. According to our previous studies^51,56^, the following steps were conducted. After noise regression, the point process analysis was adopted to detect spontaneous neural events^57^. The BOLD signals larger than mean plus one SD were detected and the onsets of neural events were extracted for HRF reconstruction^58^. The HRF in each voxel was evaluated by matching BOLD signal with the canonical HRF and its time derivative. After that, neural level signals were recovered by Wiener de-convolution (https://www.nitrc.org/projects/rshrf)^59^.

### Contributor detection for the relationship between GS fluctuations and age

**Figure 4:**
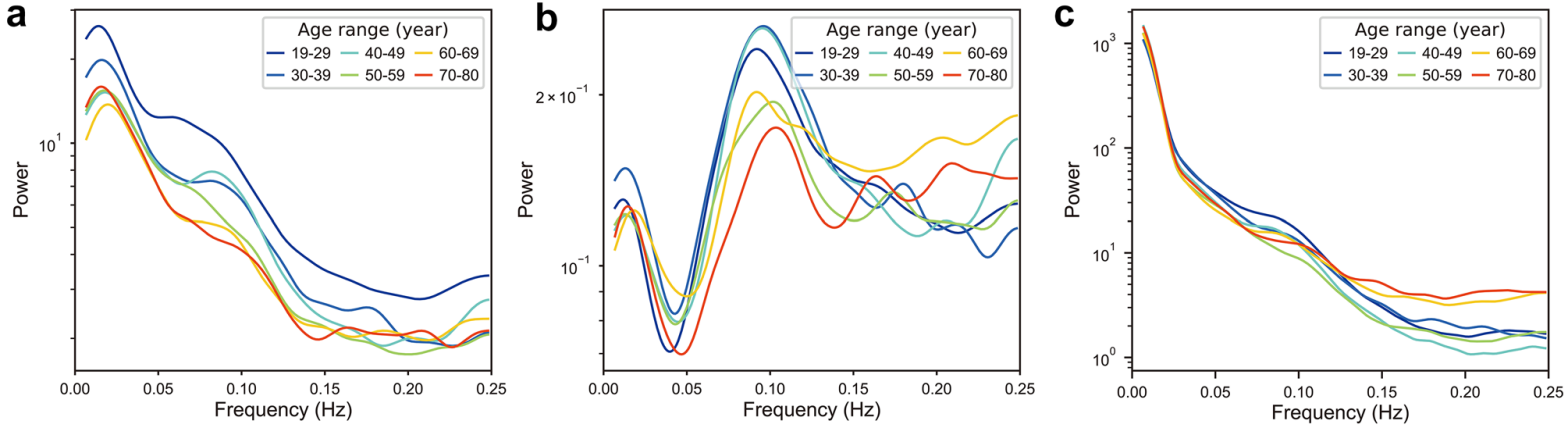
The power spectrum of GS. (**a**) Original data. (**b**) De-convolved data. (**c**) Data without noise regression. Each line represents the average power spectrum of subjects every 10 years.

The same analysis as the original data was performed for both data with de-convolution and data without noise regression to test the contribution of physiological and neurovascular coupling factors to GS fluctuations. The only difference was that the linear function *y* = *ax* + *b* was used to separate the trend from oscillations for data with de-convolution because the power-law trend disappeared after HRF de-convolution (see Fig. 4).

Using Pearson’s correlation, we evaluated the relationship between age and relative indices, including the mean and SD of GS, GS power at each frequency point, SCs of two oscillations, coefficients (*a*, *b*) of power-law and linear functions. Paired-samples t-tests (two-tailed) on SCs were performed to examine whether HRF de-convolution and noise regression changed the representative frequencies of two oscillations, respectively. Lastly, the correlation between the FD and age was calculated to evaluate the contribution of head motion to our results. Except for gender, all variables involved in the above statistics are continuous variables. Multiple-comparison corrections were conducted with the false discovery rate (FDR) method (q < 0.05).

## Data Availability

The MRI data used in this study are available to the public from the International Data-sharing Initiative (http://fcon_1000.projects.nitrc.org/indi/retro/sald.html).
